# Effect of PEEP and I:E ratio on cerebral oxygenation in ARDS: an experimental study in anesthetized rabbit

**DOI:** 10.1186/s12871-019-0782-y

**Published:** 2019-06-19

**Authors:** Federica Lovisari, Gergely H. Fodor, Ferenc Peták, Walid Habre, Sam Bayat

**Affiliations:** 1 Unit for Anesthesiological Investigations Department of Anesthesiology Pharmacology and Intensive Care, University of Geneva, Geneva, Switzerland; 2 University of Milano-Bicocca, Milan, Italy; 3 Department of Medical Physics and Informatics, University of Szeged, Szeged, Hungary; 4 Pediatric Anesthesia Unit, Geneva Children’s Hospital, Geneva, Switzerland; 5 Inserm UA7 STROBE Laboratory, University of Grenoble, Grenoble, France; 6 Department of Clinical Physiology, Sleep and Exercise, Grenoble University Hospital, Grenoble, France

**Keywords:** Mechanical ventilation, Near-infrared spectrometry, Blood flow/regional, Acute respiratory distress syndrome, Lung function, Hemodynamics

## Abstract

**Background:**

Although PEEP and inversed I:E ratio have been shown to improve gas exchange in ARDS, both can adversely affect systemic hemodynamics and cerebral perfusion. The goal of this study was to assess how changes in PEEP and I:E ratio affect systemic and cerebral oxygenation and perfusion in normal and injured lung.

**Methods:**

Eight anesthetized Chinchilla-Bastard rabbits were ventilated at baseline with pressure-regulated volume control mode, V_T_ = 6 ml/kg, PEEP = 6 cmH_2_O, FIO_2_ = 0.4; respiratory rate set for ETCO_2_ = 5.5%, and I:E = 1:2, 1:1 or 2:1 in random order. Ultrasonic carotid artery flow (CF), arterial (PaO_2_), jugular venous blood gases and near infrared spectroscopic cerebral oxygenation (∆HBO_2_) were recorded for each experimental condition. After induced lung injury, the animals were ventilated with PEEP = 9 followed by 6 cmH_2_O.

**Results:**

At baseline, inverse-ratio ventilation (IRV) significantly reduced cerebral oxygenation (∆O_2_HB; − 27 at 1:2; − 15 at 1:1 vs. 0.27 μmol/L at 2:1; *p* < 0.05), due to a significant reduction in mean arterial pressure and CF without modifying gas exchange. In injured lung, IRV improved gas exchange but decreased cerebral perfusion without affecting brain oxygenation. The higher PEEP level, however, improved PaO_2_ (67.5 ± 19.3 vs. 42.2 ± 8.4, *p* < 0.05), resulting in an improved ∆HBO_2_ (− 13.8 ± 14.7 vs. –43.5 ± 21.3, *p* < 0.05), despite a drop in CF.

**Conclusions:**

Our data suggest that unlike moderate PEEP, IRV is not effective in improving brain oxygenation in ARDS. In normal lung, IRV had a deleterious effect on brain oxygenation, which is relevant in anesthetized patients.

**Electronic supplementary material:**

The online version of this article (10.1186/s12871-019-0782-y) contains supplementary material, which is available to authorized users.

## Background

Positive-pressure mechanical ventilation is the mainstay of respiratory supportive therapy in patients with acute respiratory distress syndrome (ARDS) [1, 2]. The goal of mechanical ventilation in this setting is to open the alveoli and maintain lung aeration throughout the respiratory cycle, while avoiding excessive mechanical stretch on the lung tissue, and cyclic opening and closing of small airways and alveoli [3, 4], which can both lead to ventilator-induced lung injury. Application of positive end-expiratory pressure (PEEP) is one of the main strategies for maintaining lung aeration following alveolar recruitment [5, 6]. Another proposed approach is to increase the time during which positive pressure is applied at the airway opening, through inverse-ratio ventilation (IRV) [7–9]. This prolonged inspiratory time has been demonstrated to reduce alveolar collapse and to improve oxygenation in patients with ARDS [10, 11].

The beneficial effects of both PEEP and IRV, however, come at the potential cost of detrimental hemodynamic consequences, as the increase in intra-thoracic pressure reduces cardiac output, compromising peripheral tissue perfusion, and ultimately oxygenation [12]. Brain tissue oxygenation is directly affected by the changes in both systemic hemodynamics and blood oxygenation. It closely depends on blood perfusion and cellular metabolism in the brain, which are intricately and continuously coupled [13]. Moreover, cerebral blood flow is locally autoregulated within physiological limits of cerebral perfusion pressure [14, 15]. Available data suggest that cerebrovascular autoregulation may be impaired in a significant number of patients with ARDS [16]. Currently, it is not well understood how the potentially conflicting effects of positive pressure ventilation settings, namely PEEP and ratio of inspiratory to expiratory time (I:E), on cerebral perfusion and blood oxygenation affect brain tissue oxygenation. The importance of understanding the impact of ventilator settings on brain oxygenation is further underscored by the high prevalence of cognitive impairment in ARDS survivors [17], where both mechanical ventilation [18] and brain tissue hypoxia [19] may play a causative role.

The aim of this study was to characterize how changes in PEEP and I:E ratio affect cerebral perfusion and brain tissue oxygenation in an experimental model of ARDS in anesthetized rabbits. We hypothesized that the improvements in gas exchange by either strategy could potentially improve brain oxygenation in the presence of lung injury.

## Methods

### Ethics approval

All experiments and procedures were conducted under approval from the Swiss Animal Welfare Committee (Geneva Cantonal Veterinary Office, registration number GE/164/15).

### Animal preparation

Eight male pathogen-free Chinchilla-Bastard rabbits (3.5 ± 0.3 kg), purchased from the University of Geneva (Animalerie d’Arare, Plan-les-Ouates, Switzerland), were sedated with intramuscular Xylazine (Provet SA, Lyssach, Switzerland; 5.0 mg/kg). Following the insertion of a 22G catheter in a marginal ear vein, general anesthesia was initiated and maintained by a continuous infusion of propofol (B. Braun Medical AG, Sempach, Switzerland, 15–20 mg/kg/h) and fentanyl (Sintetica SA, Mendrisio, Switzerland; 5 μg/kg/h). All animals were intubated using a 3.0 mm cuffed endotracheal tube. Mechanical ventilation was initiated with the pressure-controlled mode using a pediatric respirator (SERVO-i, Maquet Critical Care, Solna Sweden), with, at baseline: a fraction of inspired oxygen (FiO_2_) of 0.4, a PEEP of 3 cmH_2_O and an inspiratory pressure of 6 cmH_2_O above PEEP. Tidal volume (VT) was approximately 6 ml/kg and respiratory rate (RR) was adjusted to maintain end-tidal CO_2_ (ETCO_2_) between 5.5 and 6%.

After ensuring proper depth of anesthesia, muscle relaxation was induced by a continuous infusion of atracurium (Tracrium, Labatec Pharma SA, Meyrin, Switzerland; 0.6 mg/kg/h). Fluid status was maintained by an IV infusion of lactated Ringer’s solution (Fresenius Kabi AG, Oberdorf, Switzerland; 4 ml/kg/h) after an initial bolus of 0.5 to 1.0 ml per kg of hydroxyethyl starch at the time of anesthesia induction.

The right internal jugular vein was cannulated with a 16G catheter for monitoring central venous pressure (CVP; Arrow, Teleflex Medical Europe, Westmeath, Ireland). A second 20G catheter was inserted in the left internal jugular vein cranially to allow cerebral venous blood sampling for blood gas analysis (Abbott i-Stat Handheld, Abbott Medical, Baar/Zug, Switzerland). A Doppler ring probe was positioned around the left internal carotid artery for continuous carotid blood flow (CF) measurement (Transonic Systems, Ithaca, NY). The left femoral artery was cannulated (22 G, Abbocath, Abbott Medical) for invasive blood pressure monitoring and arterial blood gas analysis. The electrocardiogram (ECG) was continuously monitored (Bio Amp, ADInstruments, Dunedin, New Zealand). The animals were placed on a thermostatic heating pad and internal body temperature was maintained at 38–39 °C (Harvard Apparatus, South Natick, MA, USA). An alveolar recruitment maneuver was then performed by inflating the lungs twice to 25 cmH_2_O for 15 s to standardize volume history. Arterial, central venous and respiratory pressure, ECG and CF signals were digitized and recorded at 1 kHz using an analog/digital interface (Powerlab model 8/35, ADInstruments). Physiological parameters were allowed to stabilize for 15 min before starting the experimental protocol.

### Brain oxygenation monitoring by near-infrared spectroscopy

Brain and muscle oxygenation was measured concomitantly to determine whether the observed changes in brain oxygenation result from local or systemic processes. Following appropriate skin shaving and cleaning, probes were placed on the head and the mid-dorsal region of the animal to monitor brain and muscle tissue oxygenation, using a NIRS monitor (NIRO-200NX, Hamamatsu, Solothurn, Switzerland). The device provides the following indices: the change in oxygenated hemoglobin (ΔHbO_2_), the change in deoxygenated hemoglobin (ΔHHO_2_), and the tissue oxygenation index (TOI), which is defined as the ratio of oxygenated to total tissue hemoglobin [20].

### Measurement of respiratory mechanics

Mechanics of airways and respiratory tissue were measured using the forced oscillation technique, as detailed previously [21]. Briefly, a loudspeaker-in-box system was used to generate a small-amplitude (1 cmH_2_O peak to peak) composite pressure forcing signal in a frequency range of 0.5–21 Hz through the tracheal cannula during short (10 s) pauses interposed with mechanical ventilation. A model including frequency-independent airway resistance (Raw) and inertance (Iaw), in series with a constant-phase tissue model [22], including damping (G) and elastance (H) was fit to the respiratory impedance (Zrs) spectra. As previously established, Raw mainly reflects the flow resistance of the airways, Iaw is related to the cyclic acceleration and deceleration of the intra-thoracic gas, G describes the energy loss within the respiratory tissues (resistance), and H characterizes the the respiratory tissues elastance. Raw and Iaw values were corrected by removing the instrumental components of the ET-cannula and the connecting tubing.

### Study protocol

The study was performed in a single group (*n* = 8), each animal serving as its own control. The experiments were carried out at an academic laboratory between approximately 8 AM and 4 PM. Fifteen minutes before baseline measurements, the ventilation mode was switched to pressure-regulated volume control mode, with a VT of 6 ml/kg, FiO_2_ of 0.4, PEEP of 6 cmH_2_O and RR set to maintain ETCO_2_ between 5.5 and 6%. The animals were then randomized to one of three different I:E ratios (1:2, 1:1 or 2:1). After 10 min with the initial I:E ratio, arterial and jugular venous blood gases and respiratory mechanics data were collected, and the I:E ratio was changed in random order following a recruitment maneuver to reset lung volume history.

ARDS was subsequently induced by a bolus intravenous lipopolysaccharide injection (from *Escherichia coli* O111:B4, 300 μg/kg, Sigma, Saint Louis, Missouri, USA) and whole lung lavage by instillation and recovery of 60 ml of 30 °C normal saline 5 times through the tracheal cannula. This was followed by injurious ventilation using volume-controlled ventilation with a VT of 10 ml/kg, a PEEP of 0, a FiO_2_ of 1.0 and I:E ratio of 1:2, until an arterial oxygen tension (PaO_2_)/fractional inspired oxygen (FiO_2_) ratio between 100 and 200 was reached (in approximately 20 to 30 min) which corresponds to moderate ARDS according to the Berlin definition [1]. Following the induction of ARDS, the same procedures performed at baseline were repeated, first at a PEEP of 9 cmH_2_O and again at PEEP of 6 cmH_2_O, and an FIO_2_ of 0.9. At the end of the study, the animal was euthanized by intravenous injection of sodium pentobarbital (120 mg/kg).

### Statistical analysis

Data are presented as mean ± standard error (SEM). Brain oxygenation indices were considered as the main outcome of the study. A formal sample size estimate could not be performed a priori since the variability of the main outcome parameter in rabbit was not known. Three-way repeated measures analysis of variance (ANOVA) with Dunnett’s post hoc tests were used to assess the effects of lung injury (control vs. injured), PEEP level (6 vs. 9 cmH2O) and I:E ratio (1:2, 1:1 and 2:1) on parameters reflecting ventilation, brain and muscle oxygenation, hemodynamics, and respiratory mechanics. The associations between hemodynamic and brain oxygenation indices were assessed with Pearson product-moment correlation tests. The statistical tests were performed within the R core package with the lme4 [23] and lsmeans [24] packages and SigmaPlot (version 12.5, Systat Software, Inc., Chicago, IL, USA) with a significance level of *p* < 0.05, and all *p* values were two-sided.

## Results

**Fig. 1:**
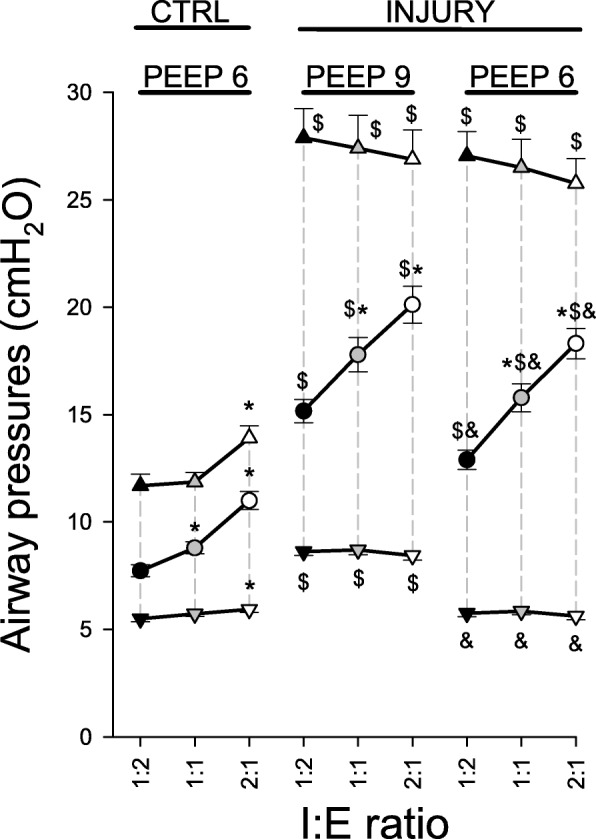
Airway pressures. CTRL: control; *triangles*: peak airway pressure; *circles*: mean airway pressure; *inverted triangles*: end-expiratory pressure (PEEP); *: vs. 1:2; $: vs. control; &: vs. injury at PEEP 9 cmH_2_O

The changes in airway pressures following the application of the different I:E ratios are shown in Fig. 1. Increasing I:E from 1:2 to 1:1 and 2:1 led to significant elevations in mean airway pressure regardless of the presence of injury (*p* < 0.001). In normal lungs, IRV resulted in both an increase in peak airway pressure (PIP, *p* < 0.05) and the appearance of a slight but significant intrinsic PEEP (*p* < 0.05). Conversely, no change in PIP and PEEP was evident in injured lungs.

**Fig. 2:**
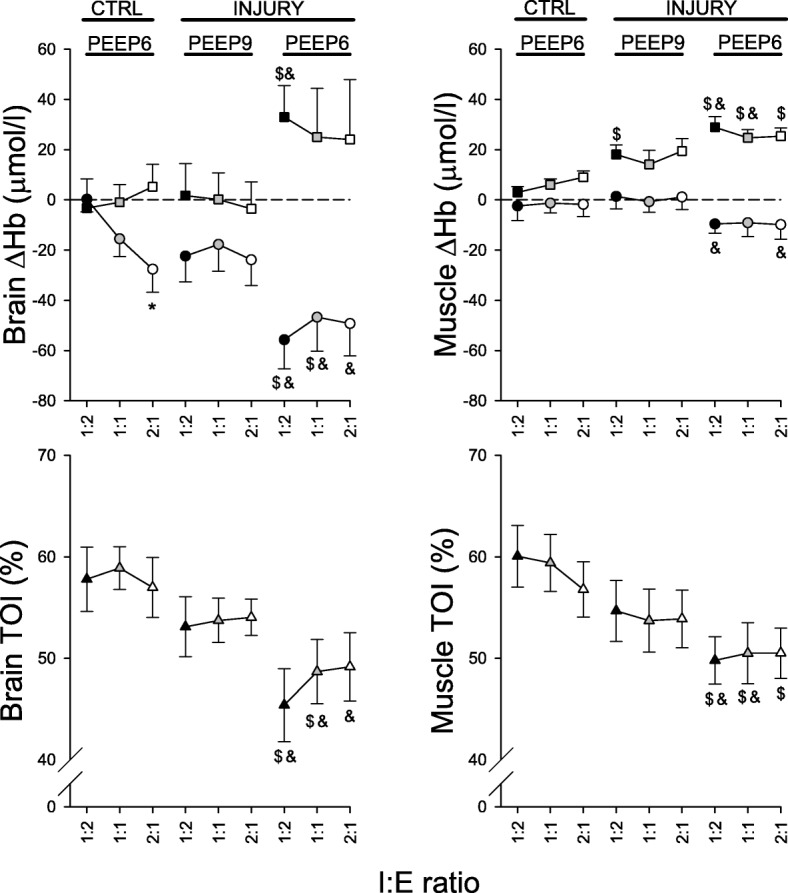
Oxygenation indices for brain and spinal muscle; ΔHb: change in hemoglobin; *circles*: change in oxygenated hemoglobin (ΔO_2_Hb); *squares*: change in deoxygenated hemoglobin (ΔHHb); *triangles*: tissue oxygenation index (TOI); *: vs. 1:2; $: vs. control; &: vs. injury at PEEP 9 cmH_2_O

Figure 2 summarizes the effects of lung injury and the changes in I:E ratio on brain and peripheral muscle tissue oxygenation as measured by NIRS. Under the control conditions, IRV led to a significant decrease in ΔO_2_Hb (*p* < 0.05), whereas all other brain and muscle oxygenation indices remained constant. Induction of lung injury resulted in significant deterioration in these indices at the lower PEEP level (*p* < 0.05). These detrimental changes were effectively counteracted at the higher PEEP level in injured lungs (*p* < 0.05).

**Fig. 3:**
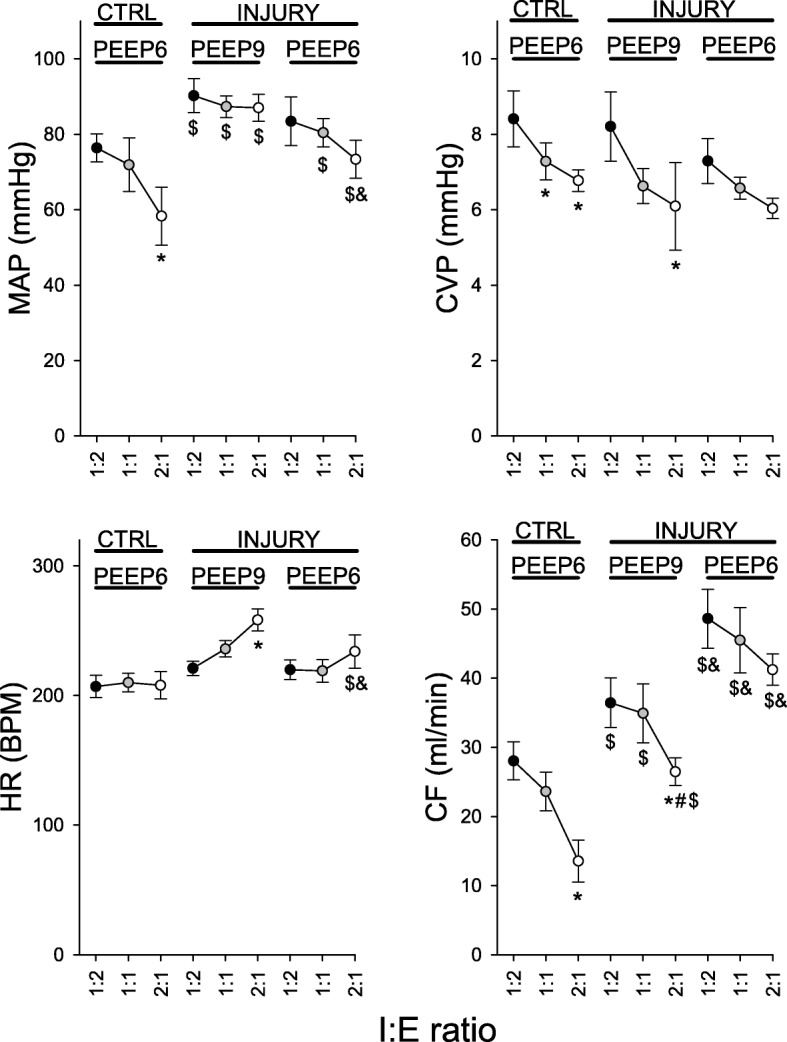
Hemodynamic parameters. CTRL: control; MAP: mean arterial pressure; CVP: central venous pressure; HR: heart rate; CF: carotid artery flow; *: vs. 1:2; #: vs. 1:1; $: vs. control; &: vs. injury at PEEP 9 cmH_2_O

Changes in systemic hemodynamics are depicted in Fig. 3. Under control conditions, increasing the I:E ratio decreased venous return as suggested by the decrease in CVP (*p* < 0.005). The MAP (*p* < 0.02) and CF (*p* < 0.001) were also decreased significantly. Following the induction of lung injury, the observed hemodynamic changes were variable and influenced by both the PEEP level and the I:E ratio. IRV in the injured lungs led to significant changes in CVP (*p* < 0.05), HR (*p* < 0.002) and CF (*p* < 0.02).

**Fig. 4:**
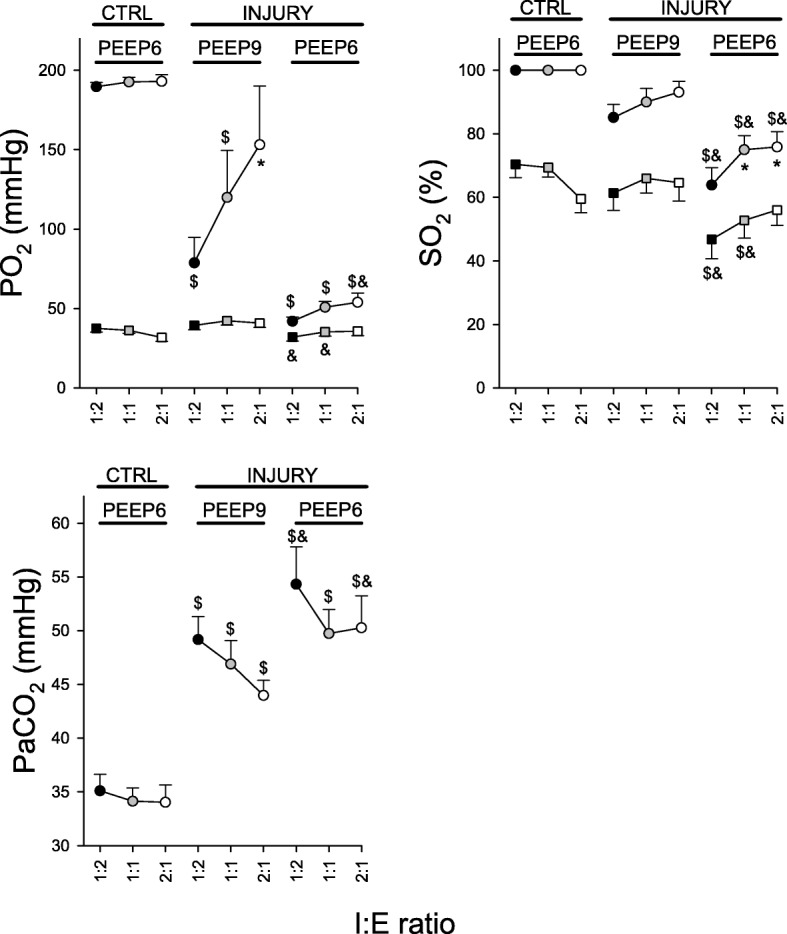
Gas exchange parameters. CTRL: control; PO_2_: oxygen partial pressure in arterial and jugular venous blood; PaCO_2_: arterial carbon dioxide partial pressure; SO_2_: oxygen saturation in arterial and jugular venous blood; *circles*: values obtained from arterial blood; *squares*: values obtained from arterial blood; *: vs. 1:2; $: vs. control; &: vs. injury at PEEP 9 cmH_2_O

Figure 4 shows the main gas exchange parameters, and a complete picture of changes in blood gas parameters and brain oxygen uptake are shown in Table 1. Lung injury compromised gas exchange significantly, which was manifested in the drop in both arterial and jugular venous blood oxygen partial pressures (*p* < 0.05). Arterial and jugular venous O_2_ (*p* < 0.001 and *p* < 0.005, respectively), CO_2_ partial pressures (*p* < 0.001 and *p* < 0.01, respectively) and O_2_ saturation (*p* < 0.001 for both) were significantly higher at the higher PEEP level of 9 cmH_2_O. Ventilation with IRV had no significant effect on arterial blood gas parameters in normal lungs. In the injured lungs however, applying an I:E ratio of 2:1 led to an improvement in PaO_2_ at both PEEP levels (*p* < 0.005).

**Table 1:** Gas exchange data

		CTRL			INJURED					
		PEEP6			PEEP9			PEEP6		
	I:E ratio	1:2	1:1	2:1	1:2	1:1	2:1	1:2	1:1	2:1
Artery	pH	7.459 ±0.02	7.467 ±0.03	7.454 ±0.02	7.244^$^ ±0.03	7.253^$^ ±0.04	7.269^$^ ±0.04	7.202^$^ ±0.04	7.221^$^ ±0.02	7.218^$^ ±0.02
	PaCO_2_ (mmHg)	35.1 ±1.54	34.1 ±1.24	34.0 ±1.60	49.2^$^ ±2.15	46.9^$^ ±2.18	44.0^*$^ ±1.39	54.3^$&^ ±3.47	49.7^$&^ ±2.21	50.3^$&^ ±2.99
	PaO_2_ (mmHg)	189.6 ±2.78	192.6 ±2.93	193 ±4.21	78.8^$^ ±16.1	119.9^$^ ±29.7	153.1^*$^ ±37.0	42.0^$&^ ±2.67	50.9^$&^ ±3.51	53.9^*$&^ ±5.82
	PaO_2_/FIO_2_	474.1 ±6.96	481.6 ±7.32	482.5 ±10.5	87.5^$^ ±17.9	133.2^$^ ±33.0	170.1^*$^ ±41.1	46.7^$&^ ±2.96	56.5^$&^ ±3.91	59.9^$&^ ±6.47
	HCO_3_^−^(mmol/l)	24.9 ±0.68	24.7 ±1.04	23.8 ±0.80	21.2^$^ ±0.54	20.8^$^ ±1.2	20.4^$^ ±1.2	21.2^$^ ±1.0	20.3^$^ ±0.5	20.3^$^ ±0.6
	BE (mmol/l)	1.0 ±0.93	0.89 ±1.42	−0.13 ±1.10	−6.4^$^ ±0.98	−6.4^$^ ±1.7	− 6.5^$^ ±1.8	−6.9^$^ ±1.6	− 7.3^$^ ±0.75	−7.5^$^ ±0.50
	SaO_2_ (%)	100.0 ±0.0	100 ±0.0	100 ±0.0	85.1^$^ ±4.2	90.0^$^ ±4.3	93.1^$^ ±3.4	63.9^$&^ ±5.4	75.0^$&^ ±4.4	75.9^$&^ ±4.8
	Lac (mmol/l)	2.67 ±0.44	2.61 ±0.43	3.07 ±0.50	4.98 ±1.0	5.03 ±1.1	5.71 ±1.1	6.36^$^ ±1.2	5.9^$^ ±0.80	5.9^$^ ±0.45
Vein	pH	7.406 ±0.016	7.412 ±0.02	7.400 ±0.02	7.227^$^ ±0.03	7.232^$^ ±0.04	7.238^$^ ±0.03	7.191^$^ ±0.04	7.197^$^ ±0.02	7.225^$^ ±0.01
	PvCO_2_ (mmHg)	38.5 ±2.0	38.2 ±1.83	39.9 ±1.85	53.7^$^ ±2.80	47.7^$^ ±2.8	49.5^$^ ±2.9	56.0^$^ ±4.7	54.8^$&^ ±2.3	52.1^$^ ±2.6
	PvO_2_ (mmHg)	37.5 ±2.3	36.3 ±1.86	31.8^*^ ±2.49	39.4 ±2.71	42.3 ±2.79	40.9^$^ ±2.92	32.0^$^ ±2.5	35.3^&^ ± 2.5	35.7^&^ ± 2.8
	HCO_3_^−^(mmol/l)	24.09 ±1.0	24.38 ±1.3	24.5 ±1.1	22.2^$^ ±0.5	20.3^$^ ±1.5	21.0^$^ ±0.96	21.1^$^ ±1.0	21.2^$^ ±0.61	21.5^$^ ±1.0
	BE (mmol/l)	−0.50 ±1.0	−0.25 ±1.5	−0.5 ±1.3	−5.5^$^ ±0.8	−7.1^$^ ±2.1	−6.4^$^ ±5.8	−7.1^$^ ±1.5	−7.0^$^ ±0.9	−6.1^$^ ±1.1
	SvO_2_ (%)	70.4 ±4.2	69.4 ±2.9	59.5^*^ ±4.3	61.4 ±5.4	66.0 ±4.7	64.6 ±5.8	46.8^$&^ ±6.1	52.8^$&^ ±5.6	56.0^$&^ ±4.8
	Lac (mmol/l)	8.41 ±0.31	8.37 ±0.33	8.19 ±0.28	8.16 ±0.28	7.63 ±0.64	7.93 ±0.31	7.71 ±0.33	8.16 ±0.31	8.27 ±0.56
	C(a-v)O_2_ (ml/dl)	3.82 ±0.57	3.91 ±0.22	4.98^*^ ±0.50	2.79^$^ ±0.31	2.66^$^ ±0.33	3.49^*$^ ±0.31	1.84^$&^ ±0.25	2.53^$^ ±0.27	2.73^*$^ ±0.48
	Brain $$ \dot{Q} $$ O_2_ (ml/min)	2.05 ±0.24	1.82 ±0.19	1.22^*^ ±0.24	1.95 ±0.17	1.82 ±0.25	1.84^$^ ±0.22	1.71 ±0.21	2.36^*^ ±0.31	2.28^*$^ ±0.40

Gas exchange and brain metabolic parameters; *BE*: base excess; *SaO*_*2*_, *SvO*_*2*_: arterial and venous hemoglobin oxygen saturation, respectivley; *Lac*: blood lactate; *C(a-v)O*_*2*_: difference in arterio-venous oxygen content; $$ \dot{Q} $$ O_2_:brain O_2_ uptake; *: vs. 1:2, $: vs. CTRL, &: vs. injury at PEEP 9 cmH_2_O; by 3-way repeated measures ANOVA

**Fig. 5:**
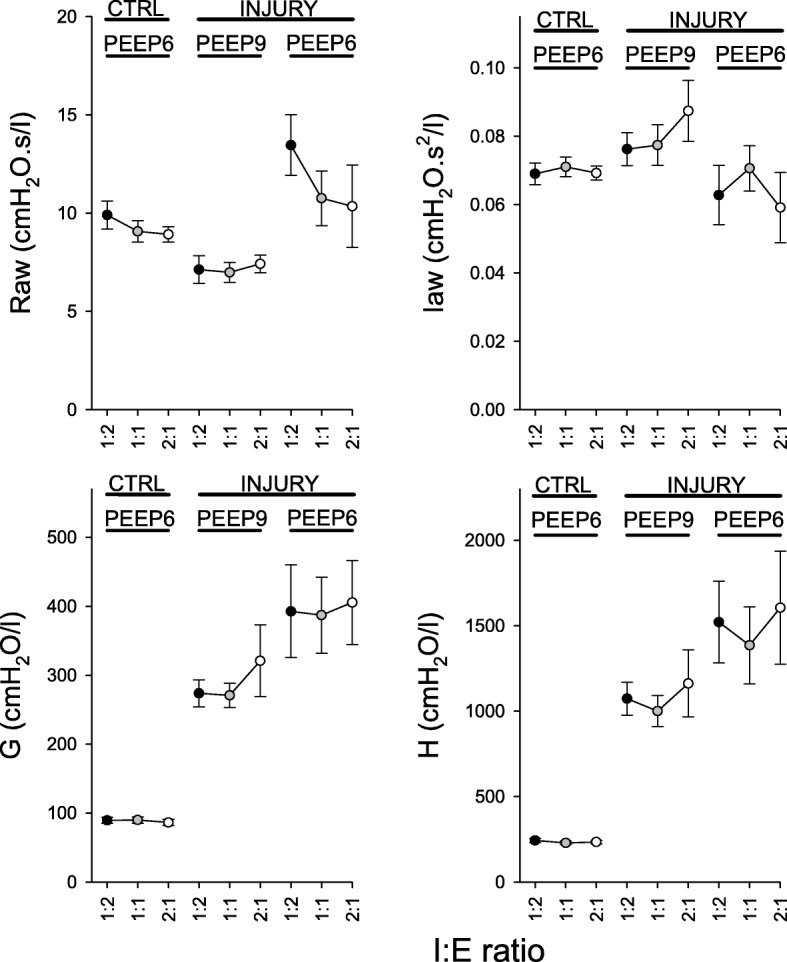
Forced oscillatory respiratory mechanical parameters; Raw: airway resistance; Iaw: airway inertance; H: respiratory tissue elastance; G: respiratory tissue damping; CTRL: control; *: vs. 1:2; #: vs. 1:1; $: vs. control; &: vs. injury at PEEP 9 cmH_2_O

Figure 5 presents the airway and respiratory tissue mechanical parameters. In the presence of the lower PEEP and a normal I:E ratio (1:2), lung injury resulted in marked and statistically significant increases in Raw (*p* < 0.002) and decreases in Iaw (*p* < 0.02). At the lower PEEP level, significant deteriorations in respiratory tissue mechanics were present in the injured lung independently of the I:E ratio applied (*p* < 0.05 for all). The Raw (*p* < 0.05), G (*p* < 0.05) and H (*p* < 0.03) were all significantly lower at the higher PEEP level of 9 cmH_2_O in injured lungs.

## Discussion

The main findings of this study were that in injured lungs, there was no evidence for an effect of IRV on brain oxygenation despite a positive effect on gas exchange. Conversely, in normal lungs, IRV led to a deterioration of brain oxygenation with a decrease in ΔO_2_Hb. This effect was consequent to the significant drop in both mean systemic arterial pressure (MAP) and CF. Unlike with IRV, brain oxygenation was significantly better with a moderate PEEP of 9 cmH_2_O as compared to a lower PEEP level of 6 cmH_2_O, in injured lungs. Overall, our data suggest that the beneficial effect of a moderate level of PEEP was more prominent than that observed with IRV.

One of the most challenging aspects of ventilating patients with ARDS is the tradeoff between maintaining optimal gas exchange with appropriate systemic hemodynamic parameters and regional tissue oxygenation. In this study, we applied a model of lung injury which reproduces the cardinal features of ARDS, with a PaO_2_/FiO_2_ < 200 [1] and a major reduction in respiratory tissue compliance. To our knowledge, despite extensive studies of the cardiorespiratory effects of IRV [25–30] no data are available on the effects of PEEP and IRV on brain oxygenation in the presence of ARDS, without concomitant brain injury.

The rationale behind the use of IRV is to improve alveolar recruitment by prolonging the time during which positive pressure is applied at the airway opening during inspiration. Lengthening inspiratory time leads to an increase in mean airway pressure, without increasing peak pressures. Therefore, increasing I:E ratio has the potential to improve the mechanical properties of the airways and respiratory tissues and gas exchange [25, 26, 28]. However, both PEEP and IRV can potentially reduce venous return due to an increase in intrathoracic pressure, thereby decreasing cardiac output [27, 28]. Both could contribute to a decrease in CF due to a reduction in effective cerebral perfusion pressure, and in cerebral oxygenation. Examination of the physiological consequences of these modes on cerebral oxygenation are therefore important and relevant. Although IRV is not widely used in clinical practice, recent studies in the literature suggest that it might be used to maintain end-expiratory recruitment without the adverse effects of PEEP [29]. Other ventilation modes have been proposed such as airway pressure-release ventilation (APRV), which is an inverse-ratio pressure controlled, intermittent mandatory ventilation, that is intensely used in some centers and used as the primary mode in ARDS [31, 32]. This mode bears similarities with IRV in that mean airway pressure is increased through a prolonged inspiratory time, without increasing peak airway pressure [33].

Inverse-ratio ventilation resulted in a net decrease in ΔO_2_Hb at baseline. In injured lung however, the effect of IRV on brain oxygenation was not significant. These changes may be attributed to the concomitant changes in gas exchange and brain perfusion as measured by CF. In turn, CF depended on MAP and was significantly influenced by PaCO_2_ (Additional file 1: Figure S1). At baseline, the drop in ΔO_2_Hb with IRV may be attributed to a decreased carotid flow, while the gas exchange parameters were unchanged. The decrease in CF under IRV in this case, may have resulted from a decreased cardiac output. This is in line with the decrease in MAP. Moreover, the decrease in CVP suggests a reduced venous return with IRV (Fig. 3). Additionally, the decrease in CF was correlated to Paw_m_ (Additional file 1: Figure S1). On the other hand, the lack of a significant effect of IRV on ΔO_2_Hb in ARDS may be due to a concomitant increase in PaO_2_ and a drop in CF (Figs. 3 and 4) at the higher PEEP level of 9 cmH_2_O.

In normal lung, the correlations between CF and brain oxygenation indices (Additional file 1: Figure S1) were opposite to those observed in moderate ARDS. This is explained by the fact that the predominant effect of an increase in Paw_m_ was a drop in CF, while gas exchange was not significantly affected (Fig. 4). Under this condition, brain oxygenation indices were primarily determined by brain perfusion, which was itself dependent upon the MAP, which is illustrated by a strong correlation between MAP and CF.

In injured lung, IRV significantly increased PaO_2_ and reduced PaCO_2_, although this effect was highly dependent on PEEP: a much larger improvement in PaO_2_ and a reduction in PaCO_2_ was observed at the higher PEEP level of 9 cmH_2_O. The lower PEEP level of 6 cmH_2_O resulted in a severe hypoxemia and hypercapnia. Despite a higher carotid flow promoted by hypercapnic cerebral vasodilation, brain oxygenation was significantly decreased as a consequence of severe hypoxemia. Under this condition, IRV had no significant effect on brain oxygenation.

A potential mechanism for the improved gas exchange on PEEP 9 cmH_2_O, may be that small increments in Paw_m_ under IRV, resulted in lung recruitment. However, although a modest increase in PEEP improved respiratory tissue elastance suggesting recruitment, no additional decreases in H were observed by increasing I:E (Fig. 5). It should be noted that IRV did not produce any intrinsic PEEP at either PEEP levels in injured lung, although a slight intrinsic PEEP was observed at baseline (Fig. 1). Nevertheless, poorly compliant alveoli with faster emptying time constants, that derecruit upon end-expiration are likely to be aerated for longer periods of time during IRV. This mechanism would contribute to gas exchange [34], but would not be measured by respiratory elastance, which is measured upon expiration. However, in injured lung IRV did not significantly increase the dynamic compliance either, despite a significant improvement with the PEEP increment from 6 to 9 cmH2O (Additional file 1: Figure S1). Our data therefore, do not demonstrate a better alveolar recruitment under IRV.

Another possible mechanism for the observed improvement in gas exchange is that the prolonged inspiration lengthens the time for admixture of alveolar and dead space gas, thereby reducing physiologic dead space [25, 27, 34]. This effect may have been more prominent in the presence of a larger alveolar gas compartment due to the recruitment induced by the slightly higher PEEP level.

Ventilating the lung with a slightly higher PEEP (9 vs. 6 cmH_2_O) with an I:E of 0.5, improved tissue oxygenation markedly in the brain and, to a lesser degree, in peripheral muscle. This effect on brain oxygenation occurred despite a significant decrease in CF (Fig. 3) that occurred in parallel to a reduction in PaCO_2_, while MAP and CVP were unchanged. This suggests that increased brain oxygenation in this case, was mainly due to improved gas exchange (Fig. 4). This interpretation is further illustrated by the negative correlation between CF and brain oxygenation indices (TOI, ΔHbO_2_) in injured lung (Additional file 1: Figure S1).

Our study had several methodological limitations. Only a single PEEP level was tested at baseline. This was because higher PEEP levels are seldom applied in normal lung, and our data show that higher airway pressures in healthy lungs can indeed compromise cerebral perfusion and systemic hemodynamics without improving gas exchange. To avoid the confounding effects of evolving lung injury and limited time, only one PEEP increment was assessed in injured lung. However, the single PEEP increment was sufficient to characterize the effect of elevated PEEP on the brain oxygenation indices; further PEEP elevations would likely produce similar trends with changes of larger magnitude. In this study, we did not alter respiratory rate in order to adjust PaCO_2_ between the various ventilatory conditions; we rather maintained the same level of external PEEP and minute ventilation, and modified the I:E ratio. While this resulted in changes in physiologic parameters with significant co-linearity amongst themselves, this is inherent to in-vivo studies in general and similar to the comparison of standard I:E and IRV in the clinical setting [27]. Moreover, manipulating the respiratory rate would have modified Paw_m_ raising further issues. We therefore maintained a study design that allows assessing the integral effect of manipulating I:E on cerebral oxygenation. We did not measure cardiac output, and it should be noted that CVP may be a weak indicator of volume status [35], although it has been shown to decrease with hypovolemia and rise with resuscitation in rabbits [36]. However, the drop in MAP is highly suggestive of a drop in cardiac output under IRV at baseline. Finally, as in any study using animal models, extrapolation to human subjects should be made with appropriate caution. However, cerebrovascular autoregulation in rabbit has been widely used as a consistent model of human physiology [37, 38].

## Conclusions

We found that the mechanical ventilation strategy in the presence of lung injury significantly impacted brain oxygenation. In normal lung, IRV had a deleterious effect on brain oxygenation, and clinicians should be aware of the potential adverse effect of this ventilation mode. Moreover, we observed no effect of IRV on brain oxygenation in moderate ARDS, as the beneficial effects of IRV on gas exchange were counteracted by decreases in cerebral perfusion. In contrast, a modest PEEP significantly improved brain oxygenation in injured lung, since the substantial improvement in gas exchange overcame the concomitant decrease in brain perfusion. Overall, our data suggest that unlike moderate PEEP, IRV is not effective in improving brain oxygenation in ARDS.

## Additional file

Additional file 1: **Figure S1.** Correlations between airway pressures and brain tissue oxygenation, perfusion, and hemo-dynamic, gas exchange parameters. (DOCX 294 kb)

## Data Availability

The datasets used and/or analysed during the current study are available from the corresponding author on reasonable request.

## Abbreviations

APRV: Airway pressure-release ventilation
ARDS: Acute respiratory distress syndrome
CF: Carotid flow
ECG: Electrocardiogram
ET-cannula: Endotracheal cannula
ETCO_2_: End-tidal CO_2_
FiO_2_: Fraction of inspired O_2_
G: respiratory tissue damping
H: respiratory tissue elastance
I:E: Ratio of inspirartory/expiratory time
Iaw: Respiratory inertance
IRV: Inverse-ration ventilation
MAP: Mean arterial pressure
NIRS: Near-infrared spectroscopy
PaO_2_: arterial O_2_ partial pressure
PEEP: Positive end-expiratory pressure
PIP: Peak inspiratory pressure
Raw: Airway resistance
RR: Respiratory rate
TOI: Tissue oxygenation index
VT: tidal volume
Zrs: respiratory impedance
ΔHbO_2_: change in oxygenated hemoglobin
ΔHHO_2_: change in deoxygenated hemoglobin
