# Supplementary material for: Effect of PEEP and I:E ratio on cerebral oxygenation in ARDS: an experimental study in anesthetized rabbit
Source: BMC Anesthesiol. 2019 Jun 19;19:110. doi: 10.1186/s12871-019-0782-y (PMC6582519; doi:10.1186/s12871-019-0782-y)
Supplement: Supplementary file 1 — Figure S1. Correlations between airway pressures and brain tissue oxygenation, perfusion, and hemo-dynamic, gas exchange parameters. (DOCX 294 kb) [file 12871_2019_782_MOESM1_ESM.docx]

**Effect of PEEP and I:E ratio on cerebral oxygenation in ARDS: An experimental study in anesthetiZed rabbits**

**SUPPLEMENTAL DIGITAL CONTENT**

Frederica Lovisari MD^1, 2^, Gergely Fodor, MD, PhD^1^, Ferenc Peták PhD, DSc^3^, Walid Habre MD, PhD^1,4^, Sam Bayat, MD, PhD^1^

^1^Unit for Anesthesiological Investigations, Department of Anesthesiology, Pharmacology and Intensive Care, University of Geneva, Geneva, Switzerland

^2^ University of Milano-Bicocca, Milano, Italy

^3^Department of Medical Physics and Informatics, University of Szeged, Szeged, Hungary

^4^Pediatric Anesthesia Unit, Geneva Children’s Hospital, Geneva, Switzerland

**Supplemental Digital Content Figure 1.** Correlations between airway pressures and brain tissue oxygenation, perfusion, and hemo-dynamic, gas exchange parameters. ΔHbO2: change in oxygenated hemoglobin; CF: carotid artery flow; MAP: mean arterial pressure; PaCO2: arterial carbon dioxide partial pressure; Paw_m_: mean airway pressure.

**Supplemental Figure 2.** Dynamic Compliance (Cdyn); CTRL: control; *: vs. 1:2; #: vs. 1:1; $: vs. control; &: vs. Injury at PEEP 9 cmH_2_O.
